# *Burkholderia cepacia* infection in children without cystic fibrosis: a clinical analysis of 50 cases

**DOI:** 10.3389/fped.2023.1115877

**Published:** 2023-05-15

**Authors:** Huixuan Shi, Xianrui Chen, Lili Chen, Bizhen Zhu, Weiyuan Yan, Xiaobo Ma

**Affiliations:** ^1^ Department of Pediatrics, The First Affiliated Hospital of Xiamen University, Xiamen, China; ^2^ Pediatric Key Laboratory of Xiamen, Xiamen Science and Technology Bureau, Xiamen, China; ^3^ Institute of Pediatrics, School of Medicine, Xiamen University, Xiamen, China; ^4^ Department of Pediatric Rehabilitation, Xiamen Rehabilitation Hospital, Xiamen, China; ^5^ Department of Clinical Laboratory, The First Affiliated Hospital, School of Medicine, Xiamen University, Xiamen, China; ^6^ Xiamen Key Laboratory of Genetic Testing, Xiamen Science and Technology Bureau, Xiamen, China; ^7^ School of Public Health, Xiamen University, Xiamen, China

**Keywords:** *Burkholderia cepacia*, opportunistic pathogen, hospital infection, child, antimicrobial susceptibility, pediatric disease

## Abstract

**Background:**

*Burkholderia cepacia* (*B. cepacia*) is an emerging pathogen of nosocomial infection in pediatric patient carrying cystic fibrosis. The clinical diagnosis and treatment of *B. cepacia* infection remains poorly studied. This study outlined the risk factors, antimicrobial susceptibility, and clinical characteristics aiming to improve the treatment of *B. cepacia* infection.

**Methods:**

A retrospective study was conducted based on the 50 cases infection caused by *B. cepacia* in children without cystic fibrosis, which were diagnosed in the First Affiliated Hospital of Xiamen University, from January 1st, 2011 to December 31st, 2021.

**Results:**

A total of 50 children were infected with *B. cepacia*, of whom 68% had an underlying health condition, such as cardiovascular disease (23.5%), respiratory disease (17.6%), nervous system disease (14.7%), and neoplastic disease (14.7%). At the onset of *B. cepacia* infection, 42 (84%) pediatric patients were in an intensive care unit (ICU), 33 (66%) underwent endotracheal intubation, and 32 (64%) had a central venous catheter (CVC). In addition, hospital-acquired cases were 46 (92%), and healthcare-acquired cases were 4 (12%). The most common infectious sites of *B. cepacia* were the respiratory tract (68%), followed by the blood (20%), and the urinary tract (12%). It indicated that *B. cepacia* was the most sensitive to ceftazidime (95.65%), followed by trimethoprim-sulfamethoxazole (88.68%), meropenem (82.98%), cefepime (77.78%), and levofloxacin (55.85%). The drug resistance rate of piperacillin-tazobactam, minocycline, aztreonam, cefoperazone-sulbactam and ceftriaxone was higher than 55%. 38 cases were cured or improved, eight had treatment terminated, and four died.

**Conclusion:**

*B. cepacia* is an opportunistic pathogen normally found in immunocompromised pediatric patients and highly likely to lead to drug resistance. Nosocomial *B. cepacia* infections occurred mostly in patients in the ICU based on our observations. The surveillance of *B. cepacia* infections including changing epidemiology and increasing resistance of the microorganism is still very important. Treatment with effective antibiotics such as ceftazidime, meropenem, trimethoprim-sulfamethoxazole is associated with a favorable prognosis.

## Introduction

*Burkholderia cepacia complex (Bcc)* is a group of Gram-negative, catalase-producing, glucose-nonfermenting, obligately aerobic bacilli composed of phylogenetically closely related bacterial species, at least 20 different types, which is identified by the *Burkholderia cepacia* recA gene sequences (1). *Burkholderia cepacia* (*B. cepacia*) is an opportunistic pathogen that can cause severe infections in patients with underlying diseases, such as cystic fibrosis (CF) or chronic granulomatous disease (CGD) (2). *B. cepacia* has been reported to cause nosocomial bloodstream infections in non-CF patients with a mortality rate of 25%–64% (3). Emerging amounts of clinical publications have attempted to demonstrate the clinical manifestation and management of pediatric *B. cepacia* infection. Several severe infections caused by *B. cepacia* have been reported in children, including sepsis, pneumonia, CNS infections, and urinary tract infections (4, 5). Our study was performed to explore the risk, antimicrobial susceptibility, and clinical performance of *B. cepacia* infection, aiming to improve the strategies for treatment and prognosis.

## Materials and methods

### Study design and setting

Based on a retrospective review of the historical pediatric cases hospitalized in the First Affiliated Hospital of Xiamen University from January 1st, 2011 to December 31st, 2021.

### Population

A total of 50 pediatric cases accepted by the pediatric department of the First Affiliated Hospital of Xiamen University were diagnosed with *B. cepacia* infection.

### Inclusion/exclusion criteria

The inclusion criteria were: (1) patients under 14 years old, (2) Positive *B. cepacia* cultures of body fluid samples, central venous catheter tips, or endotracheal tube tips. The following criteria were excluded: the patient had a definitive cystic fibrosis diagnosis.

### Samples and data collection

In accordance with the Manual of Clinical Microbiology (6, 7), the following body fluid samples were collected: endotracheal aspirates, deep sputum, bronchoalveolar lavage (BAL), blood (from peripheral veins or central venous catheters), cerebrospinal fluid, and urine. Moreover, demographics, clinical and laboratory data (prognosis, complications) were documented for each patient.

### Definitions of infections

Infections caused by nosocomial bloodstream bacteria were defined by the presence of a positive blood culture in patients who had been hospitalized for more than 48 h. A patient was considered to have a health care-associated bloodstream infection if a positive blood culture was observed from the patient at the time of hospital admission or within 48 h if any of the following conditions were fulfilled: (1). The 30 days before the bloodstream infection, the patient received home intravenous therapy, wound care or specialized nursing care from a health care agency, family, or friends. Patients whose only home therapy was oxygen use were excluded. (2). Attended a hospital or hemodialysis clinic or received intravenous chemotherapy in the 30 days before the bloodstream infection. (3). Was hospitalized in an acute care hospital for two or more days in the 90 days before the bloodstream infection. (4). Resided in a nursing home or long-term care facility.

Community-acquired bloodstream infection was defined by a presence of positive blood culture observed at the time of hospital admission or within the 48 h after admission. Patients meeting the criteria will be considered as community associated infection (8).

In addition, definite catheter-related bloodstream infection was defined as a presence of positive blood culture, along with a presence of positive catheter tip culture yielding the same species of microorganism, or the growth of the same pathogen from blood cultures of the CVC and a peripheral vein, with positive values for the differential time to positivity (9).

Community-acquired pneumonia (CAP) is an infection acquired outside of a hospital. Infection acquired after at least 48 h in the hospital is defined as hospital-acquired pneumonia (HAP). Ventilator-associated pneumonia (VAP) is defined as a subcategory of HAP that occurs in patients receiving mechanical ventilation. Infection acquired in low-acuity healthcare settings such as nursing homes and dialysis centers is known as healthcare-associated pneumonia (HCAP) (10).

### Microbiologic identification

Samples were inoculated onto eosin methylene blue agar (Autobio Diagnostics Co Ltd.) and Colombian blood agar medium (Autobio Diagnostics Co Ltd.). All inoculated plates were incubated in Thermo M3111 incubator (Thermo Inc.). Pathogen identifications were performed using the Vitek MS-CHCA (BioMérieux Inc.) and Vitek-MS automated microbial identification system (BioMérieux Inc.).

Antimicrobial susceptibility testing of *B.cepacia* isolates were determined by the automated VITEK 2 compact microbiology analyzer (BioMérieux Inc., France). Results were interpreted as sensitive, intermediate and resistance based on the Clinical and Laboratory Standards Institute's (CLSI) criterias (11–21). For statistical analysis purpose, “intermediate” sensitivity results of bacterial isolates were grouped to “resistant” sensitivity results. The quality control strains of pathogen identification were *E.coli ATCC 25922* strains (National Center for Clinical Laboratories, China) and *E.coli ATCC 8739* strains (Biomerieux Inc., France). For quality control of susceptibility tests, Pseudomonas aeruginosa ATCC 27853 strains (National Center for Clinical Laboratories, China) were used.

BioMerieux mini Vidas automated immunoassay analyzer (BioMérieux Inc.) and procalcitonin (PCT) kit were used to detect serum PCT. C-reactive protein (CRP) was detected by VITROS 5,1 FS analyzer (Ortho-Clinical Diagnostics) using the manufacturer's reagents.

### Statistical analysis

Statistical analyses were performed using SPSS software (version 22.0; IBM SPSS, Inc., Chicago, IL, USA). Discrete numbers and percentages were used to represent categorical variables. A mean and standard deviation (SD) were used to present continuous variables. T test was used to conduct a statistical comparison, with significance determined by a *p* value < 0.05.

In this study, the baseline characteristics, clinical characteristics, laboratory findings and antimicrobial susceptibility were investigated in 50 cases of *B. cepacia* infection. Furthermore, we summarized empirical antibiotic treatments and antibiotic treatments after receiving an antibiogram. The prevalence and prognosis of infections caused by *B. cepacia* was investigated among non-CF pediatric patients with or without the underlying disease.

## Results

### Patient characteristics

During the study period, 50 pediatric patients were diagnosed with *B. cepacia* infection, 18 (36%) of whom were female and the median age was 15 months (one day, 123 months). In this patient cohort, 68% had underlying diseases. The most common underlying disease was cardiovascular disease (23.5%), followed by respiratory diseases (17.6%), nervous system disease (14.7%) and neoplastic disease (14.7%). There were six patients with respiratory diseases, including two cases of tracheomalacia and tracheal stenosis, and one case of idiopathic pulmonary fibrosis and pulmonary alveolar proteinosis. One patient with tracheomalacia was tracheotomy dependent. Patients with idiopathic pulmonary fibrosis and pulmonary alveolar proteinosis were both treated with long-term oral prednisone. There were five patients with neurological disorders, including one each with spinal muscular atrophy and mitochondrial encephalomyopathy, central hypoventilation syndrome, Charcot-Marie-Tooth disease, intracranial hemorrhage with Ommaya capsule implantation. At the onset of infection with *B. cepacia*, these five patients were intubated and ventilated. The demographic characteristics of the patients were listed in Table 1 below.

**Table 1:** Demographic characteristics of 50 children with *B. cepacia* infection.

Characteristic	Value
Age, median (Interquartile range)	15 months (1 day, 123 months)
Female gender *n* (%) of patients	18 (36%)
Underlying diseases *n* (%) of patients	34 (68%)
Cardiovascular disease	8 (23.5%)
Respiratory diseases	6 (17.6%)
Nervous system disease	5 (14.7%)
Neoplastic disease	5 (14.7%)
Urinary tract malformation	4 (11.7%)
Multiple trauma or drowning	3 (8.8%)
Chronic granulomatous disease	2 (5.9%)
End-stage renal disease	1 (2.9%)
**Underlying conditions *n* (%) of patients**	
ICU stay at onset of infection	42 (84%)
Presence of endotracheal intubation	33 (66%)
Presence of central venous catheter	32 (64%)
Using mix antibiotics	23 (46%)
Presence of urethral catheter	20 (40%)
Previous chemotherapy or immunosuppressant use	16 (32%)
Previous surgery within 1 month	6 (12%)
Newborn (premature)	4 (8%)

ICU, intensive care unit.

The clinical characteristics of 50 pediatric cases having *B. cepacia* infection were tabulated in Table 2. Upon onset of *B. cepacia* infection, 42 (84%) children were in the ICU, 33 (66%) underwent endotracheal intubation, and 32 (64%) received a central venous catheter. Four cases (8%) were health care-associated infections, and 46 (92%) were hospital-acquired infections. Infection with *B. cepacia* most commonly occurs in the respiratory tract (68%), blood (20%), and urinary tract (12%).

**Table 2:** Clinical characteristics of 50 children with *B. Cepacia* infection.

Characteristic	Value
**Mode of acquisition, *n* (%) of patients**	
Health care–associated	4 (8%)
Hospital-acquired	46 (92%)
**Culture-positive specimen, *n* (%) of patients**	
Endotracheal aspirate or deep sputum	24 (48%)
Bronchoalveolar lavage fluid	10 (20%)
Blood	10 (20%)
Urine	6 (12%)
Central venous catheter tip	4 (8%)
**Infection site, *n* (%) of patients**	
Pneumonia	34 (68%)
Urinary tract infection	6 (12%)
Bloodstream infection (CVC-related infection, combined with pneumonia, Unknown origin)	10 (4,4,2)(20%)

### Laboratory findings

Pediatric patients with *B. cepacia* infection had elevated CRP and PCT levels, as well as an increase in neutrophils. A statistically significant difference between patients with and without underlying diseases was not found in the experimental results shown in Table 3.

**Table 3:** The laboratory findings of patients.

Laboratory indexes	Total (*n* = 50)	With underlying diseases (*n* = 28)	Without underlying diseases (*n* = 22)	*p*
WBC count (10^9^/L)	12.05 ± 6.5	11.90 ± 6.54	12.24 ± 6.61	0.857
Neutrophil (%)	61.78 ± 17.79	59.22 ± 15.83	65.05 ± 19.90	0.255
Hemoglobin (g/L)	102.78 ± 25.28	101.00 ± 18.21	105.05 ± 32.48	0.580
Platelet count (10^9^/L)	332.1 ± 247.88	373.86 ± 277.47	278.95 ± 197.74	0.182
CRP (mg/L)	31.27 ± 28.46	25.94 ± 30.63	35.03 ± 26.23	0.320
PCT (ng/ml)	3.55 ± 8.68	3.38 ± 8.31	3.70 ± 9.23	0.915

Reference intervals were the following: CRP (0–10 mg/L), PCT (<0.046 ng/ml).

### Antimicrobial susceptibility

The antimicrobial susceptibility testing results of *B. cepacia* isolated were shown in Table 4. It was found that 95.65% and 88.68% of *B. cepacia* isolates were susceptible to ceftazidime and trimethoprim-sulfamethoxazole (TMP-SMX). Susceptibility rates for meropenem, cefepime, and levofloxacin were 82.98%, 77.78%, 53.85%, respectively. Isolates of *B. cepacia* showed low susceptibility to cefoperazone-sulbactam (22.22%), ceftriaxone (22.22%), tigecycline (20%), and ticacillin/potassium clavulanate (1%). A susceptibility test revealed 100% resistance to nitrofurantoin and tobramycin.

**Table 4:** Results of antimicrobial susceptibility testing of *B. Cepacia* isolates from 50 patients.

Antibiotics	Sensitive (%)	Intermediate (%)	Resistant (%)
Ceftazidime (*n* = 46)	44 (95.65)	2 (4.35)	/
TMP-SMX (*n* = 53)	47 (88.68)	/	6 (11.32)
Meropenem (*n* = 47)	39 (82.98)	4 (8.51)	4 (8.51)
Cefepime (*n* = 18)	14 (77.78)	2 (11.11)	2 (11.11)
Levofloxacin (*n* = 52)	28 (53.85)	6 (11.54)	18 (34.62)
piperacillin tazobactam (*n* = 34)	15 (44.12)	1 (2.94)	18 (52.94)
Minocycline (*n* = 14)	6 (42.86)	3 (21.43)	5 (35.71)
Aztreonam (*n* = 17)	6 (35.29)	4 (23.53)	7 (41.18)
cefoperazone-sulbactam (*n* = 9)	2 (22.22)	3 (33.33)	4 (44.44)
Ceftriaxone (*n* = 18)	4 (22.22)	1 (5.56)	13 (72.22)
Tigecycline (*n* = 10)	2 (20)	3 (30)	5 (50)
Ticacillin/potassium clavulanate (*n* = 24)	1 (4.17)	/	23 (95.83)
Nitrofurantoin (*n* = 19)	0	1 (5.26)	18 (94.74)
Tobramycin (*n* = 19)	0	/	19 (100)

### Treatment and outcome

The empirical antibiotic treatments and adjustment of antibiotics after the antibiogram available were shown in Table 5. Empiric antibiotic regimens were adjusted in 30 cases. The most common empiric antibiotic used was cefoperazone-sulbactam (30%), followed by meropenem (28%), cefepime (8%) and piperacillin-tazobactam (8%). Meropenem was the most effective empiric antibiotics used in this research. Meropenem was used in 20 cases of patients (15 cases used alone, three cases combined with ceftazidime, one case combined with tigecycline, one case combined with TMP-SMX). In our study, five cases were treated with meropenem in combination with other antibiotics. Of all these five treatments were started with meropenem, and the second antibiotic was added according to the result of drug susceptibility test when fever persisted and clinical situation of patient continued worsening after 3 days' meropenem therapy. Only one out of the five strains was intermediate to meropenem and the others were sensitive to meropenem. In this group of 20 patients, 13 improved, four gave up treatment, and four died. The other antibiotics used after the available antibiogram were piperacillin tazobactam (12%), cefepime(10%), TMP-SMX (8%), cefoperazone-sulbactam (8%), ceftazidime (6%) ceftriaxone (4%), levofloxacin (2%) and others (8%) presented in Table 6.

**Table 5:** Empirical antibiotic treatments and adjustment of antibiotics after the antibiogram was available.

	Drug sensitivity results	Adjustment of antibiotics after the antibiogram was available	With underlying diseases	Clinical cure
**Empiric antibiotic regimens were adjusted in 30 cases**				
9 cases	Sensitive	One case was adjusted to other sensitive antibiotic due to antibiotic allergy. Four cases were adjusted to other sensitive antibiotics after five days' ineffective treatment. Four cases combinated with other sensitive antibiotics after three days' ineffective treatment.	6	7
5 cases	Resistant	Adjusted to other sensitive antibiotics	4	3
2 cases	Intermediate	Adjusted to other sensitive antibiotics	1	2
14 cases	No done	13 cases were adjusted to other sensitive antibiotics after five days' ineffective treatment. One case combinated with other sensitive antibiotics after three days' ineffective treatment.	8	11
**Empirical antibiotics (*n* = 50)**				
Cefoperazone-sulbactam (*n* = 15)	No done (*n *= 13) Resistant (*n* = 1) Intermediate (*n* = 1)	12 cases were adjusted to other sensitive antibiotics due to ineffective treatment. Three cases retained the same treatment.	8	13
Meropenem (*n* = 14)	Sensitive (*n* = 10) Resistant (*n* = 2) Intermediate (*n *= 2)	Four cases (two cases each of resistant and intermediate) were adjusted to other sensitive antibiotics due to ineffective treatment. Four meropenem-sensitive cases were combinated with other sensitive antibiotics after three days' ineffective treatment. Seven cases retained the same treatment.	7	8
Cefepime (*n* = 4)	No done (*n* = 2) Sensitive (*n* = 2)	Two cefepime-sensitive cases were combinated with other sensitive antibiotics after five days' ineffective treatment. Two cases retained the same treatment.	3	4
TMP-SMX (*n* = 2)	Sensitive (*n* = 2)	Retained the same treatment.	2	2
Piperacillin tazobactam (*n* = 4)	No done (*n* = 1) Sensitive (*n* = 2) Resistant (*n* = 1)	One case retained the same treatment. Three cases were adjusted to other sensitive antibiotics due to ineffective treatment.	3	2
Ceftriaxone (*n* = 3)	No done (*n* = 1) Sensitive (*n* = 2)	Two ceftriaxone-sensitive cases were retained the same treatment. One case was adjusted to other sensitive antibiotics due to ineffective treatment.	3	2
Ceftazidime (*n* = 2)	Sensitive (*n* = 2)	One case was adjusted to other sensitive antibiotic due to antibiotic allergy. The other case was adjusted to other sensitive antibiotics after five days' ineffective treatment.	1	1
Others (*n* = 6)	No done (*n* = 1) Resistant (*n* = 5)	Two cases were adjusted to other sensitive antibiotics due to ineffective treatment. Four cases retained the same treatment.	1	6

Ineffective treatment: fever persisted and clinical situation of patient continued worsening after 3–5 days' antibiotic therapy.

**Table 6:** Antibiotic treatments after the antibiogram was available.

Antibiotics	With underlying diseases (%)	Without underlying diseases (%)	Clinical cure (%)
Meropenem (*n* = 15)	9 (60)	6 (40)	10 (66.67)
Piperacillin-tazobactam (*n* = 6)	4 (66.67)	2 (33.33)	5 (83.33)
Cefepime (*n* = 5)	3 (60)	2 (40)	3 (60)
TMP-SMX (*n* = 4)	3 (75)	1 (25)	4 (100)
cefoperazone-sulbactam (*n* = 4)	1 (25)	3 (75)	4 (100)
ceftazidime (*n* = 3)	2 (66.67)	1 (33.33)	2 (66.67)
ceftriaxone (*n* = 2)	2 (100)	0	1 (50)
levofloxacin (*n* = 1)	1 (100)	0	1 (100)
Imipenem and Cilastatin Sodium (*n* = 1)	0	1 (100)	1 (100)
Mezlocillin-sulbactam (*n* = 1)	0	1 (100)	1 (100)
cefmetazole (*n* = 1)	0	1 (100)	1 (100)
Piperacillin-tazobactam combination with levofloxacin (*n* = 1)	1 (100)	0	1 (100)
Meropenem combination with Tigecycline (*n* = 1)	0	1 (100)	1 (100)
Meropenem combination with ceftazidime (*n* = 3)	1	2 (66.67)	2 (66.67)
Meropenem combination with TMP-SMX (*n* = 1)	1 (100)	0	0
Cefoperazone-sulbactam combination with TMP-SMX (*n* = 1)	0	1 (100)	1 (100)

Almost all patients had good responses to definitive antibiotic therapy. Furthermore, eight patients withdrew from treatment because their caregivers couldn't afford the out-of-pocket cost burden. The average length of hospital stay of 50 patients was 34.6 days, and the average length of ICU stay for 41 patients was 26.8 days. After treatment, 38 patients improved, eight patients were loss to follow-up, and four patients died. Two CGD patients both had *B. cepacia* bloodstream infection died of multiple organ failure without the presence of macrophage activation syndrome/hemophagocytic lymphohistiocytosis (MAS/HLH). One case of severe viral encephalitis died of multiple organ failure. After intracranial hemorrhage during extracorporeal membrane oxygenation (ECMO) treatment, one patient with severe adenovirus pneumonia died of cerebral herniation, as shown in Table 7.

**Table 7:** Clinical characteristics and outcomes of 50 children with *B. cepacia* infection.

	Cure	Deaths	Withdrew treatment
With underlying diseases (*n* = 28)	19	3	6
Without underlying diseases (*n* = 22)	19	1	2
Total (*n* = 50)	38	4	8

## Discussion

In healthy individuals, *B. cepacia* rarely causes infection, but can lead to life-threatening infections in those with underlying diseases such as cystic fibrosis, oncological conditions, or CGD (22). In pediatric patients over the past 11 years, *B. cepacia* infection was rare. There were only 50 non-CF patients diagnosed with *B. cepacia* infection, and two of them had CGD. 33 (68%) patients had underlying diseases, the most common being cardiovascular (23.5%), respiratory (17.6%) and nervous system (14.7%) diseases. According to the study by Kim et al., *B. cepacia* might cause hospital infections in immunocompromised children and in previously healthy pediatric patients admitted to the intensive care unit (23). Our study included 16 patients (32%) who were immunosuppressed due to chemotherapy or immunosuppressants, and 46 cases of hospital-acquired infections (including 42 cases contracted during ICU stays) and four cases of health care-associated infections.

Previous investigations had revealed invasive procedures as risk factors for *B. cepacia* infections, including CVC, hemodialysis, multiple bronchoscopies, mechanical ventilator or tracheostomy use, and recent surgery (24). In a neonatal intensive care unit, central venous catheters have been found to be a significant risk factor for *B. cepacia* nosocomial infection (25). In our study, endotracheal intubation accounted for 66% of invasive procedures, followed by CVC (64%), urethral catheter (40%), and previous surgery (12%), in addition, 50% of CVC-related bloodstream infections were reported, compared to 42% in previous research (26).

It has been reported that *B. cepacia* causes various infections in children, including bacteremia, pneumonia, urinary tract infection, endocarditis, meningitis, and brain abscess^.^(22, 27, 28). According to Peng F et al.'s study, respiratory tract infections (15/16) were the most common, followed by blood infections (5/16) (29). In a study by Tugba et al., 37% of children presented with bacteremia, and 25.9% with pneumonia (including ventilator-associated pneumonia) (4). As a result of our study, the most frequent isolation of *B. cepacia* culture was the respiratory tract secretions (68%), followed by the blood (20%) and urinary tract (12%). It was reported that *B. cepacia* was the second most prevalent organism isolated from CGD patients with bacteremia in the United States and was responsible for nearly 20% of the deaths from CGD (29). In CGD patients, 14 cases of MAS/HLH were described, four of which were triggered by a member of the *B. cepacia* complex (30). Both CGD patients in our study died due to multiple organ failure and septic shock as a result of *B. cepacia* bloodstream infections without MAS/HLH.

It has been shown that *B. cepacia* pathogens are intrinsically resistant to numerous antibiotic classes, including ampicillin, amoxicillin, piperacillin, ticarcillin, amoxicillin-sulbactam, amoxicillin-clavulanate, ertapenem, polymyxin B, colistin, aminoglycosides, and fosfomycin (31). Based on our study, *B. cepacia* was highly resistant to piperacillin-tazobactam, aztreonam, cefoperazone-sulbactam, and ceftriaxone. Tugba et al. reported that 78.2% of *B. cepacia* were resistant to piperacillin-tazobactam in pediatric infections (4). As per the Clinical and Laboratory Standards Institute guidelines, there is insufficient clinical evidence to confirm the intrinsic resistance to antibiotics, including piperacillin-tazobactam, ceftriaxone, cefepime, aztreonam, and imipenem (31). Chun-Hsing Liao et al. reported that the percentages of patients receiving piperacillin–tazobactam (*n* = 37) were similar in survivors and non-survivors with *B. cepacia* bacteremia (32). In our study, six patients received piperacillin–tazobactam treatment and five patients were cure.

According to the Sanford guide to antimicrobial therapy, the recommended antimicrobial agents against *B. cepacia* were levofloxacin, TMP-SMX, meropenem, ceftazidime, and minocycline (33). According to a Korean study (23), the antibiotic susceptibility rates of *B. cepacia* were compared to our results including meropenem (78.57% vs. 82.98%), TMP-SMX (71.43% vs.88.68%), minocycline (66.67% vs. 42.86%), ceftazidime (64.29% vs. 95.65%), and levofloxacin (50% vs. 55.85%). A study by Chun-Hsing Liao and colleagues found that *B. cepacia* isolates were sensitive to meropenem (100%), ceftazidime (97.3%), levofloxacin (5.5%) and minocycline (5.5%). The antimicrobial treatment for *B. cepacia* infection found favorable outcomes in 89.6% of patients treated with ceftazidime, 100% with meropenem (32). TMP-SMX and ceftazidime have been shown to reduce mortality in previous studies (32, 34). Favorable outcome in 65% of 20 patients treated with meropenem was reported in our study. Four patients treated with TMP-SMX were all cured. There is some hurdle to the use of TMP-SMX, since allergic or hypersensitivity reactions, intolerance (35). The use of some recommended drugs are limited due to the side effects. Use of minocycline below the age of eight is not recommended due to the potential for tooth discoloration, dental enamel hypoplasia and bone growth inhibition (36). The use of levofloxacin in children is limited due to levofloxacin-induced musculoskeletal adverse drug events (37). Ceftazidime has been suggested for the treatment of *B. cepacia* infection. The susceptibility tests in the present study showed most of the isolates were sensitive to ceftazidime, but only six patients received ceftazidime treatment. Four of six patients were cure and two gave up treatment. Treatment strategies should be proposed according to clinical condition and the antibiotic susceptibility results.Clinical pharmacist plays an important role in pharmaceutical care of anti-infective target therapy.

## Limitations

There were several deficiencies in this study. The study involved a limited number of pediatric patients with *B. cepacia* infection over a eleven-year period in a single center. As a result, the study findings may be less robust to some extent. A second limitation of our retrospective study was the limited amount of information on manifestations. As a result, our findings may not apply to CF patients, since our study included only non-CF patients.

## Conclusion

The majority of nosocomial *B. cepacia* infections occur in ICU children with underlying diseases, immunosuppressed states, or invasive procedures. A multidrug resistance issue makes *B. cepacia* a clinical treatment challenge. Therefore, it is crucial to monitor *B. cepacia* infections, including changing epidemiology and increasing resistance. A favorable prognosis can be gained by monitoring infections and using effective antibiotics such as ceftazidime, meropenem, trimethoprim-sulfamethoxazole.

## Data Availability

The original contributions presented in the study are included in the article further inquiries can be directed to the corresponding author.
